# Drug use patterns and factors related to the use and discontinuation of medications for opioid use disorder in the age of fentanyl: findings from a mixed-methods study of people who use drugs

**DOI:** 10.1186/s13011-023-00538-x

**Published:** 2023-05-22

**Authors:** Jaclyn M. W. Hughto, Abigail Tapper, Sabrina S. Rapisarda, Thomas J. Stopka, Wilson R. Palacios, Patricia Case, Joseph Silcox, Patience Moyo, Traci C. Green

**Affiliations:** 1 Department of Behavioral and Social Sciences, Brown University School of Public Health, Providence, RI USA; 2 Department of Epidemiology, School of Public Health, Brown University School of Public Health, Providence, RI USA; 3 Center for Promotion and Health Equity, Brown University, Providence, RI USA; 4 The Fenway Institute, Fenway Health, Boston, MA USA; 5 Opioid Policy Research Collaborative, The Heller School for Social Policy and Management, Brandeis University, Waltham, MA USA; 6 School of Criminology and Justice Studies, University of Massachusetts Lowell, Lowell, MA USA; 7 Department of Public Health and Community Medicine, Tufts University School of Medicine, Boston, MA USA; 8 Bouve College of Health Sciences, Northeastern University, Boston, MA USA; 9 Department of Health Services, Policy and Practice, Brown University School of Public Health, Providence, RI USA; 10 Center for Gerontology and Healthcare Research, Brown University, Providence, RI USA; 11 Division of General Internal Medicine Research, Department of Community Health, Warren Alpert School of Medicine, Providence, RI USA; 12 Department of Sociology, University of Massachusetts Boston, Boston, MA USA

**Keywords:** Medications for opioid Use Disorder, Methadone, Buprenorphine, Naltrexone, Drug Use, Overdose, Fentanyl, Cocaine, Crack, Pain Medication, Benzodiazepines

## Abstract

**Background:**

Medications for opioid use disorder (MOUD; methadone, buprenorphine, naltrexone) are the most effective treatments for OUD, and MOUD is protective against fatal overdoses. However, continued illegal drug use can increase the risk of treatment discontinuation. Given the widespread presence of fentanyl in the drug supply, research is needed to understand who is at greatest risk for concurrent MOUD and drug use and the contexts shaping use and treatment discontinuation.

**Methods:**

From 2017 to 2020, Massachusetts residents with past-30-day illegal drug use completed surveys (N = 284) and interviews (N = 99) about MOUD and drug use. An age-adjusted multinomial logistic regression model tested associations between past-30-day drug use and MOUD use (current/past/never). Among those on methadone or buprenorphine (N = 108), multivariable logistic regression models examined the association between socio-demographics, MOUD type; and past-30-day use of heroin/fentanyl; crack; benzodiazepines; and pain medications. Qualitative interviews explored drivers of concurrent drug and MOUD use.

**Results:**

Most (79.9%) participants had used MOUD (38.7% currently; 41.2% past), and past 30-day drug use was high: 74.4% heroin/fentanyl; 51.4% crack cocaine; 31.3% benzodiazepines, and 18% pain medications. In exploring drug use by MOUD history, multinomial regression analyses found that crack use was positively associated with past and current MOUD use (outcome referent: never used MOUD); whereas benzodiazepine use was not associated with past MOUD use but was positively associated with current use. Conversely, pain medication use was associated with reduced odds of past and current MOUD use. Among those on methadone or buprenorphine, separate multivariable logistic regression models found that benzodiazepine and methadone use were positively associated with heroin/fentanyl use; living in a medium-sized city and sex work were positively associated with crack use; heroin/fentanyl use was positively associated with benzodiazepine use; and witnessing an overdose was inversely associated with pain medication use. Many participants qualitatively reported reducing illegal opioid use while on MOUD, yet inadequate dosage, trauma, psychological cravings, and environmental triggers drove their continued drug use, which increased their risk of treatment discontinuation and overdose.

**Conclusions:**

Findings highlight variations in continued drug use by MOUD use history, reasons for concurrent use, and implications for MOUD treatment delivery and continuity.

## Background

Since the beginning of time, humans have used psychoactive drugs (herein referred to as drugs) such as opioids and stimulants to treat their pain, improve their mood, change their consciousness, and cope with life difficulties [1, 2]. In the case of opioids, many individuals have and continue to safely use prescribed synthetic opioid pain medications (e.g., fentanyl, oxycodone) for short-term pain management [2, 3]. However, the over-prescribing of opioid pain medications in the United States (US) and the subsequent overdose deaths and regulations to reduce the availability of these prescribed drugs has led many individuals who become dependent on prescription opioids to seek out lower-cost and widely accessible heroin and now illegally-manufactured fentanyl (herein referred to as fentanyl) on the street [4–6]. The rising use of heroin, coupled with the growing presence of fentanyl in the heroin and broader illegal drug supply, has led to an epidemic of fentanyl-involved overdose deaths in the United States [7], particularly in Massachusetts, which ranked 17th in the nation for fatal opioid-involved overdose deaths as of 2020 [8].

Medications for opioid use disorder (MOUD) – methadone, buprenorphine/ buprenorphine-naloxone (e.g., Subutex©/Suboxone©), and naltrexone (e.g., Vivitrol©) – are the most effective, evidence-based approaches to treating Opioid Use Disorder (OUD), a highly prevalent, chronic, and relapsing disease [9, 10]. MOUD works to normalize brain chemistry, block the euphoric effects of opioids, relieve physiological cravings, and normalize bodily functions for people with OUD [10, 11]. Extensive research finds that when taken as prescribed and maintained, MOUD effectively reduces opioid withdrawal, opioid use, and mortality [10, 12–15]. To that end, a retrospective chart review of people who used fentanyl and initiated methadone found that the majority (89%) of those who remained in treatment for six months achieved abstinence from fentanyl within one year, with no deaths occurring while on methadone. However, 64% of those who initiated any type of MOUD in a national study [16] and 32% of those initiating methadone in Rhode Island [14] discontinued treatment before six months, with two of those in the Rhode Island study dying within five weeks of MOUD discontinuation [14]. Although ongoing IM fentanyl use is common among people on MOUD, particularly in the first six months of treatment [14, 17], research shows that patients on MOUD who intentionally or unintentionally use fentanyl are less likely to die from a fentanyl overdose than those who discontinue treatment [14, 15, 17].

Although MOUD represents the most effective treatment for OUD [9], and has great utility for overdose mortality prevention [14, 15, 17, 18], some people on MOUD do not cease their use of illegal opioids, may abstain for a period of time and later relapse, or concurrently use other illegal and prescribed drugs, with a number of potential health and treatment-related consequences [14, 17, 19–23]. One study exploring the presence of various drugs in 200 urine samples collected from patients receiving methadone or buprenorphine found that nearly half of the samples tested positive for illegal drugs in a variety of different combinations, including benzodiazepines, amphetamines, and opioids [24]. Another study found that approximately one in eight people receiving buprenorphine prescriptions across eight US states were simultaneously prescribed benzodiazepines [25], despite the increased risk of overdose when the two drugs are combined [18, 26, 27]. Although MOUD is protective against fatal overdose, the co-use of illegal fentanyl or heroin, prescribed or street-acquired (diverted or counterfeit) pain medication and benzodiazepines is associated with increased risk of emergency department admission and excess morbidity and mortality [18, 27, 28]. Further, concurrent MOUD and illegal drug use can pose a risk for treatment disengagement as some providers may terminate MOUD for patients who routinely test positive for illegal opioids and other drugs [29, 30], in turn, increasing their risk for fatal and non-fatal overdose [14, 17, 31].

Despite the documented health risks of concurrent drug use while on MOUD, research on the factors that drive ongoing drug use among people on MOUD is limited in the literature. One qualitative research study with 26 people on methadone or buprenorphine found that participants reported using benzodiazepines with and without a prescription to manage their untreated mental health symptoms [32]. In another qualitative study of 46 people in New York City with a lifetime history of using prescription opioids for non-medical reasons, participants reported using benzodiazepines to both enhance the high of opioids and to self-manage their mental health concerns such as anxiety [33]. Additionally, a qualitative study of 39 methadone patients in Vancouver, Canada found that individuals may use cocaine to mitigate the sedative effects of methadone [34]. Research also finds that some people may psychologically crave the desire to get high, which in some cases can lead them to terminate their use of MOUD, particularly partial (buprenorphine-naloxone) and full (naltrexone) opioid antagonists, which block the euphoric effects of opioids [35]. While these studies offer some insights into the reasons why people on MOUD use stimulants, benzodiazepines, and other drugs, mixed-methods research is needed to understand the unmet treatment needs of people on MOUD in the age of fentanyl so that treatment models for OUD and co-occurring health conditions can be tailored appropriately.

In order to fill the aforementioned gaps in the literature, we first sought to: [Aim 1] use quantitative data to understand the socio-demographic characteristics and drug use patterns of the sample according to whether they had never used, previously used, or were currently using MOUD. The quantitative data were then supplemented with qualitative data to contextualize why and which individuals elected not to use, to use, or to discontinue the use of prescribed MOUD. Next, we restricted the quantitative data to only those who were currently using MOUD to: [Aim 2] identify the socio-demographic characteristics and drug use behaviors associated with the use of four prevalent drugs (heroin/fentanyl; crack cocaine; benzodiazepines; pain medications) while on MOUD. We then used qualitative data to assess the context and drivers surrounding participants’ decision to use or modify their drug use patterns while on MOUD. By exploring the patterns and contexts surrounding past and concurrent MOUD and drug use, we can help identify individuals at risk for adverse MOUD treatment outcomes and modify treatment approaches to support individual recovery goals, and reduce drug use-related morbidity and mortality among people who use drugs.

## Methods

This study is a secondary analysis of data drawn from seven Massachusetts communities sampled via a larger study. Although the methods of the parent study are described in depth elsewhere [36], briefly, a mixed-methods rapid assessment of consumer knowledge was conducted in Massachusetts between 2017 and 2019. Informed by fatal overdose trends shown in the 2015 to 2017 Massachusetts State Unintentional Drug Overdose Reporting System (SUDORS) data [37], the parent study focused on understanding risk and protective factors for overdose among 305 people who use drugs [38] in seven communities comprised of 15 cities or towns. These cities and towns were selected as they had some of the highest prevalence estimates of fatal overdoses in the state between 2015 and 2017: Chicopee; Cape Cod (Barnstable, Dennis, Falmouth, Mashpee, Orleans, Truro); Lowell, New Bedford, North Shore (Beverly, Lynn, Peabody, Salem); Quincy; and Springfield. Individuals were eligible for the study if they were 18 years of age or older; a resident of one of the seven communities in Massachusetts; and reported using an illegal drug in the past 30 days.

### Recruitment

In preparation for recruitment, we conducted environmental scans comprised of a review of publicly-available public health and surveillance data, community walk-throughs, and meetings with community partners (i.e., harm reduction organizations, community health centers, shelters, food banks, and other social service organizations). Recruitment strategies varied by study location though all strategies employed purposive sampling to ensure that we sampled individuals at highest risk of experiencing a fatal overdose based on the SUDORS data for each community. This approach prioritized the recruitment and enrollment of participants who were diverse in terms of age, race/ethnicity, primary drug of choice, and neighborhood of residence.

In Lowell, Quincy, Cape Cod, Springfield, Chicopee, and the North Shore, we used convenience sampling [39, 40]. For this approach, we primarily relied on the direct referral of participants from community partners. We also posted flyers online (e.g., Craigslist) and handed out and posted flyers at community organizations, in public spaces, and in neighborhoods where people who use drugs spend time (as determined via environmental scans and SUDORS data). Snowball sampling strategies were also utilized. Participants received $5 for up to three people whom they referred and who were eligible and enrolled in the study.

In New Bedford, we piloted the use of respondent-driven sampling (RDS) to augment recruitment [41] and assess the feasibility of this method to recruit people who use drugs. For this approach, we worked with community partners to identify “seed” participants that were at higher risk of fatal overdose according to the New Bedford SUDORS data (e.g., transactional sex workers, fishermen/anglers, people who use cocaine). We surveyed seed participants and then gave them three time-limited referral coupons for eligible “sprout” participants. Eligible sprouts who returned their referral coupon were subsequently enrolled and completed data collection. Sprout participants then received three coupons to refer new sprouts. Participants who successfully recruited others were compensated with $5 per eligible recruit (up to three per person).

### Data collection

Potential participants were screened for eligibility by phone or in person. Verbal informed consent was obtained from all participants before initiating study procedures. All participants completed a one-time, interviewer-administered survey, which lasted approximately 45 min and was carried out on a tablet or on paper. The survey assessed participants’ socio-demographic characteristics, history of personally experienced and witnessed overdoses, patterns of drug use, and MOUD history. Following the survey, approximately one-third of all participants (n = 113) completed an in-depth qualitative interview covering similar topics that were assessed in the survey. Survey participants were invited to complete an interview if they demonstrated (via their survey responses) a willingness to discuss their substance use history and related experiences and/or they reported unique or extensive drug use patterns, experiences of witnessed or personal overdose, experiences accessing harm reduction and treatment services, or other self-reported data that would enable the researchers to better contextualize the risk and protective factors for overdose beyond the data provided by the survey. The interviews were audio-recorded. The majority of the surveys and interviews were conducted in English; a subset was conducted in Spanish. Participants received $20 for each portion of the visit that they completed (i.e., survey and interview). The study was approved by the Boston University Medical Center Institutional Review Board.

### Measures

All survey questions were developed for the RACK study. Items were initially generated by the investigators based on the literature, and then pilot-tested and refined prior to data collection.

*Socio-demographics.* Age was assessed categorically and collapsed into a ternary variable of 18 to 30 years of age, 31 to 40 years of age, and 41 years of age or older. Gender categories included male, female, or transgender. Race and Hispanic ethnicity were assessed independently and combined to create the following racial/ethnic categories: White, non-Hispanic; Black, non-Hispanic; Native American, non-Hispanic; Hispanic; more than one race/ethnicity; and another race/ethnicity.

We identified the 2017 population sizes of the cities and towns where participants resided and categorized participants as living in a large city (pop size > 150,000; Springfield), medium city (pop size between 60,000 and 149,999; Lowell, Lynn, New Bedford, Quincy), and small city or town (pop size < 60,000; Barnstable, Beverly, Chicopee, Dennis, Falmouth, Mashpee, Orleans, Peabody, Salem, Truro). Employment was categorized as employed or unemployed. Participants were asked to indicate where they were currently living. Individuals who reported living in a house or apartment that they or someone else owns or rents were coded as being stably housed. Individuals who reported living on the street or in a temporary living situation such as “couch surfing,” a shelter, or the hallway of a house were coded as being unstably housed. Participants were also asked if they have ever traded sex for money, food, drugs, or other items (yes/no) and if they had a doctor or healthcare provider whom they see regularly (yes/no). Additionally, the number of times that participants had personally experienced and witnessed an overdose and whether they had overdosed in the past year were also assessed.

*MOUD Use*. Participants were also asked about their current and lifetime use of prescribed MOUD (yes/no), including methadone, buprenorphine (i.e., buprenorphine or buprenorphine-naloxone), and naltrexone. A categorical variable of current, past, and no history of any MOUD use was also created.

*Drug Use.* Participants were asked whether they had ever received (i.e., bought, traded, given/gifted) a fake pain pill on the street (yes/no). Pain medication access was assessed by asking participants to report how hard it is to obtain a genuine prescription pain pill on the street compared to last year (easier, about the same, harder) and how hard it is to obtain a prescription for opioid pain medication from a doctor (extremely hard, hard, neither, easy, extremely easy).

Participants were also asked to report their drug use history, with the four most prevalent drugs used in the past 30 days presented: heroin/fentanyl, crack cocaine, benzodiazepines (street-acquired or prescribed), and pain medications (street-acquired or prescribed). Since most drugs bought as heroin or “dope” in Massachusetts contain fentanyl, we combined heroin and fentanyl as a single variable.

### Data analysis

All quantitative analyses were conducted in SAS 9.4. Individuals who did not have a history of opioid use and those with missing data for MOUD history were excluded, leaving an analytical sample of N = 284. Additional missing data are reported in Table 1. None of the variables included in the final multivariable models had missing data.

**Table 1:** Characteristics of the sample among people who use drugs in Massachusetts (N = 284)

SOCIO-DEMOGRAPHICS	N	%
**Age**		
18–30	71	25.0
31–40	104	36.6
41+	109	38.4
**Gender**		
Female	111	39.1
Male	173	60.9
**Race/Ethnicity**		
White, non-Hispanic	188	66.2
Person of color	96	33.8
**Geographic Location**		
Large city	42	14.8
Medium city	166	58.5
Small city or town	76	26.8
**Employment Status**		
Employed	125	44.0
Unemployed	159	56.0
**Housing Status**		
Stable housing	144	50.7
Unstable housing	140	49.3
**History of Sex Work (n = 235)**		
No	158	67.2
Yes	77	32.8
**Has a Regular Healthcare Provider (n = 283)**		
No	91	29.9
Yes	192	63.2
**Number of Times Witnessed an Overdose**	**Mean**	**SD**
IQR: 13	12.7	18.6
**Number of Times Overdosed**		
IQR: 4	4.0	9.2
**Overdosed - Past Year**	**N**	**%**
No	168	59.2
Yes	116	40.8
**DRUG USE & AVAILABILITY**		
**Received Fake Pain Pill on Street (n = 255)**		
No	133	52.2
Yes	122	47.8
**Ease of Getting a Prescription Pain Pill on Street - Currently Compared to Previous Year (N = 224)**		
Easier	36	15.6
About the same	57	24.7
Harder	131	46.1
**Ease of Getting a Prescription Pain Pill from Doctor - Current (N = 231)**		
Extremely Easy	4	1.7
Easy	17	7.4
Neutral	15	6.5
Hard	89	38.5
Extremely Hard	106	45.9
**Number of Years Using Illegal Drugs**	**Mean**	**SD**
IQR: 18	18.5	9.7
**Heroin/Fentanyl Use - Past 30 Days**	**N**	**%**
No	73	25.7
Yes	211	74.3
**Crack Cocaine Use - Past 30 Days**		
No	138	48.6
Yes	146	51.4
**Benzodiazepine Use - Past 30 Days**		
No	195	68.7
Yes	89	31.3
**Pain Medication Use - Past 30 Days**		
No	233	82.0
Yes	51	18.0
**MOUD USE**		
**Any MOUD Use (N = 284)**		
Never	57	20.1
Currently	110	38.7
In the Past	117	41.2
**Methadone Use - Lifetime (n = 281)**		
No	143	50.9
Yes	138	49.1
**Buprenorphine Use - Lifetime (n = 282)**		
No	107	37.9
Yes	175	62.1
**Naltrexone Use - Lifetime (n = 284)**		
No	227	79.9
Yes	57	20.1
**Types of MOUD Used - Currently (n = 110)**		
Methadone	46	41.8
Buprenorphine	62	56.4
Naltrexone	2	1.8

Using the full analytic sample (N = 284), multinomial logistic regression analysis (Model 1) was used to explore the associations between the four most commonly used drugs in the past 30 days (heroin/fentanyl [yes/no]; crack cocaine [yes/no]; benzodiazepines [yes/no]; pain medications [yes/no]) and the Model 1 Outcome: prescribed MOUD in the past (referent = never) and currently (referent = never). Since the goal of this analysis was to explore how drug use differs by MOUD history, we did not explore the association between socio-demographic characteristics and the outcome. However, since older individuals have more years to use MOUD than younger individuals, we adjusted for age.

Among the subset of participants on methadone or buprenorphine (n = 108), bivariate logistic regression analyses were used to examine the association between socio-demographics (age, gender, race/ethnicity, city, employment status, housing status, history of sex work, access to a regular healthcare provider, number of overdosed experienced and witnessed); past 30-day drug use; MOUD type (buprenorphine or methadone) and four drug-use outcomes: heroin/fentanyl; crack cocaine; benzodiazepines; and pain medication use – all in the past 30 days. Participants on naltrexone were excluded from the multivariable logistic regression analyses due to the small size of this group (n = 2). The independent variables were selected based on their availability in the parent study and evidence from the qualitative data and prior literature linking specific characteristics and behaviors to drug use among people on MOUD. Factors associated with each outcome at *p* < 0.10 in the bivariate analyses were entered into the respective multivariable model. Since MOUD use and drug use preferences may vary by age, we adjusted for age in all multivariable models regardless of significance in bivariate analyses. For all analyses, significance was determined at *p* < 0.05.

Qualitative interviews were recorded and transcribed verbatim by a professional transcription service. The transcripts were first reviewed by the research team for content and clarity. The transcripts were then imported into NVivo 12, a qualitative data analysis program, and subsequently analyzed using an inductive and deductive approach. In preparation for analysis, an initial codebook was created using the key thematic areas contained in the interview guide, including drug use and MOUD history. The interview transcripts were then reviewed and open-coded for emerging themes and subthemes. Through a series of team meetings and ongoing transcript reviews, emerging themes were integrated into the codebook. Using NVivo, two trained research assistants with expertise in qualitative research coded the transcripts using a rapid, first-cycle coding approach [42]. A total of 25% of the transcripts were double-coded to ensure consistency in the coding application. The coders met weekly with the first author to review the application of codes and to revise the codebook, code definitions, and coding application as necessary. After completing the initial rapid coding process, the first and second authors applied a second layer of codes pertinent to the present quantitative analysis (e.g., reason for concurrent drug and MOUD use, rationale for the use of specific MOUD types). The coded transcripts from the 99 participants with a past or current history of MOUD use were then used to contextualize the findings of the quantitative analyses.

## Results

### Socio-Demographics

The majority of the sample (61.6%) was age 40 or younger, male (60.9%), White, non-Hispanic (66.2%), and came from a medium-sized city (58.5%) (Table 1). About half of the sample was unemployed (56.0%) and had unstable housing (49.3%), yet approximately two-thirds of participants (63.2%) reported having a doctor they saw regularly. Of the 235 participants who were asked about their history of sex work, 32.8% reported having ever engaged in sex work. On average, participants reported witnessing an overdose 12.7 times (SD = 18.6) and personally overdosing 4.0 times (SD = 9.3). Two-fifths of the sample (40.8%) had overdosed in the past year.

### MOUD use

As shown in Table 1, the majority (79.9%) of people who use drugs in this sample had received MOUD in their lifetime (41.2% in the past; 38.7% currently) and 20.1% had never used MOUD. In considering lifetime use, 62.1% had taken buprenorphine, 49.1% methadone, and 20.1% naltrexone. For those currently prescribed MOUD (n = 110), 56.4% were on buprenorphine, 41.8% methadone, and 1.8% naltrexone.

Qualitative interviews contextualized the patterns of MOUD use and preferences for specific types. For example, although all participants in this sample reported using one or more illegal drugs in the past 30 days, many participants found MOUD to be effective in reducing their opioid withdrawal symptoms and cravings and reducing their use. For example, one male participant from a medium city indicated that being prescribed MOUD allowed him to reduce his use of illegal opioids, noting, “*So I didn’t get sick. The methadone took my cravings away and I wasn’t getting high on it*.” Similarly, another female participant from a large city noted, “*Oh, when I stopped [using heroin]…I didn’t even go back, not even once. I started the [buprenorphine] program, and I’m still in the program. I don’t crave it*.”

In discussing preferences for MOUD type via qualitative interviews, many people favored buprenorphine over methadone, which aligns with the higher prevalence of past 30-day buprenorphine use reported by participants in the survey relative to other types of MOUD. In fact, some participants reported switching to buprenorphine from methadone as it did not produce a high or make them as tired as methadone and allowed them to achieve some of their recovery goals. One female participant on buprenorphine from a medium city noted: *“[People still try to get high on [methadone] and they actually get high on it and that’s why I chose to do the Suboxone… it helps you get through the day, you’re not sick in the morning when you wake up, and it doesn’t have the same side effects as the methadone*.” Similarly, one male participant noted:

*I used to like the methadone better because at the time, my mentality, even though I got on it to get clean, the methadone would give you this high. That even when you’re sober you kind of feel some kind of high on it to be honest with you. So, I liked it for the longest time. The reason I choose Suboxone now is because Suboxone give me more energy. It don’t get me high. I feel normal. And it give me energy, and my plan is to start working, get a job. I don’t know, for some reason the way methadone hits me, I always want to, I take, like, three naps a day, like I’m always tired. So, the Suboxone don’t get me like that. I feel like I never touched drugs before. Like, normal. –***Currently Prescribed Buprenorphine, Medium City**

Notably, only two of the people who use drugs in this sample reported being on naltrexone, and only 20% reported a lifetime history of naltrexone use. As a full opioid antagonist, naltrexone blocks the euphoric effects of opioids and leads to withdrawal for those who attempt to use opioids [10]. In qualitative interviews, the efficacy of naltrexone in reducing cravings and preventing opioid use was specifically cited by some participants as a rationale for taking it. Though for some, the inability to concurrently use illegal opioids while on naltrexone led them to choose methadone or buprenorphine over naltrexone. For example, one male participant noted:

*The Vivitrol, I loved it. Oh, yeah. Full of energy. It was probably one of the best things I ever did. I had no urge [to use heroin] at all. ..[But] I got complacent. You know what I mean? I wanted to do things my way again….the Suboxone you administer it yourself. So if I felt like getting high one day, I just went…and just got high. If you’re sick, then you can just take a Suboxone*. *–***Formerly Prescribed All Types of MOUD, Medium City**

Although some participants found naltrexone to be the most effective medication for discontinuing their use of opioids, some also felt that medication alone was insufficient to manage their untreated trauma, and so they were driven to continue using heroin/fentanyl and MOUD simultaneously to cope with the psychological toll of past trauma. For example, one female participant reported:

*But as far as an opioid blocker, [Vivitrol] worked. But, like I said, that wasn’t the cure all for me. Like, it’s not just like, I can get Vivitrol and be fucking great….You gotta work on yourself. There’s so many other things that come into play…You want people to [get help for]...trauma or things that have happened in their lives…like, so many things come into play. –***Formerly Prescribed Naltrexone, Small City or Town**

### Drug use & MOUD history

#### Drug use & MOUD history: heroin/fentanyl

Among the full sample, heroin/fentanyl was the most used drug in the past 30 days (69.4%; Aim 1: Table 1). In the age-adjusted multinomial logistic regression model (Aim 1: Table 2) examining the association between current drug use and history of MOUD use among the full sample (N = 284), the association between past 30-day heroin/fentanyl use and past MOUD use (ref: never) was marginally significant (aOR = 1.90; 95% CI = 0.90–4.02; *p* = 0.09), and there was no significant association between past 30-day heroin/fentanyl use and current MOUD use (*p* = 0.31). When restricting the sample to only those on methadone or buprenorphine (N = 108), the multivariable logistic regression model (Aim 2: Table 3) showed that past 30-day benzodiazepine use (aOR = 3.27; 95% CI = 1.11–9.65; *p* = 0.03) and current methadone use (ref: buprenorphine use; aOR = 6.61; 95% CI: 2.03–21.56; *p* = 0.002) were each associated with increased odds of past 30-day heroin/fentanyl use.

**Table 2:** Multinomial age-adjusted logistic regression analyses examining the association between the use of heroin/fentanyl, crack cocaine, benzodiazepines, pain medication, and use in the past 30 days and prescribed medications for opioid use disorder (MOUD) among people who use drugs in Massachusetts (N = 284)

	PRESCRIBED MOUD						
	In the Past vs. Never (Referent)			Currently vs. Never (Referent)			
	aOR	95% CI	P-Value	aOR	95% CI	P-Value	
**DRUG USE - PAST 30 DAYS**							
**Heroin/Fentanyl**							
No	1.00	----	----	1.00	----	----	
Yes	1.90	0.90–4.02	0.09	1.48	0.69–3.19	0.31	
**Crack Cocaine**							
No	1.00	----	----	1.00	----	----	
Yes	2.73	1.36–5.46	**0.005**	3.05	1.49–6.26	**0.002**	
**Benzodiazepine**							
No	1.00	----	----	1.00	----	----	
Yes	1.41	0.64–3.10	0.40	2.71	1.22–6.01	**0.01**	
**Pain Medication**							
No	1.00	----	----	1.00	----	----	
Yes	0.43	0.19–0.98	**0.04**	0.31	0.13–0.73	**0.008**	

aOR = Adjusted Odds Ratio; CI = Confidence Intervals; P = P-Value; Bolded values = significant at p < 0.05.

*Note.* All variables were significantly associated with the outcome in bivariate analyses. Since older individuals have more years to use drugs and MOUD than younger individuals, the multivariable model shown here adjusted for age.

**Table 3:** Multivariable logistic regression analyses examining the association between socio-demographics and the four most prevalent drugs used in the past 30 days among individuals prescribed methadone or buprenorphine for opioid use disorder in Massachusetts (N = 108)

DRUG USE – PAST 30 DAYS																								
	HEROIN/FENTANYL						CRACK COCAINE						BENZODIAZEPINE						PAIN MEDICATION					
	aOR	95% CI	P	aOR	95% CI	P	OR	95% CI	P	aOR	95% CI	P	OR	95% CI	P	aOR	95% CI	P	OR	95% CI	P	aOR	95% CI	P
**SOCIO-DEMOGRAPHICS**																								
**Age**																								
18–30	1.00	---	---	1.00	---	---	1.00	---	---	1.00	---	---	1.00	---	---	1.00	---	---	1.00	---	---	1.00	---	---
31–40	0.75	0.20–2.79	0.66	0.58	0.13–2.54	0.47	3.71	1.17–11.80	**0.03**	2.72	0.72–10.34	0.14	1.91	0.62–5.89	0.26	2.31	0.69–7.30	0.17	0.57	0.13–2.42	0.44	0.62	0.14–2.85	0.54
41+	0.78	0.22–2.78	0.70	1.00	0.25-4.00	0.995	2.26	0.76–6.68	0.14	1.58	0.45–5.51	0.47	0.78	0.26–2.36	0.67	1.06	0.70–7.70	0.93	0.60	0.15–2.33	0.46	0.70	0.17–2.99	0.63
**Gender**																								
Male	1.00	---	---	---	---	---	1.00	---	---	---	---	---	1.00	---	---	---	---	---	1.00	---	---	---	---	---
Female	1.95	0.79–4.85	0.15	---	---	---	1.82	0.84–3.97	0.13	---	---	---	0.92	0.43-2.00	0.84	---	---	---	1.67	0.57–4.87	0.35	---	---	---
**Race/Ethnicity**																								
White, non-Hispanic	1.00	---	---	---	---	---	1.00	---	---	---	---	---	1.00	---	---	1.00	---	---	1.00	---	---	---	---	---
Person of color	1.06	0.41–2.75	0.90	---	---	---	0.71	0.31–1.62	0.41	---	---	---	0.45	0.19–1.10	*0.08*	0.51	0.18–1.43	0.20	1.04	0.33–3.27	0.95	---	---	---
**City Type**																								
Large city	2.95	0.56–15.6	0.20	---	---	---	1.93	0.55–6.71	0.53	2.16	0.49–9.54	0.31	1.93	0.55–6.79	0.31	---	---	---	0.39	0.04–3.63	0.41	---	---	---
Medium city	1.39	0.54–3.60	0.49	---	---	---	2.28	0.95–5.45	*0.06*	2.89	1.04–8.03	**0.04**	1.63	0.66–4.03	0.29	---	---	---	1.06	0.33–3.41	0.92	---	---	---
Small city or town	1.00	---	---	---	---	---	1.00	---	---	1.00	---	---	1.00	---	---	---	---	---	1.00	---	---			
**Employment Status**																								
Employed	1.00	---	---	---	---	---	1.00	---	---	---	---	---	1.00	---	---	---	---	---	1.00	---	---	---	---	---
Unemployed	0.83	0.31–2.22	0.71	---	---	---	2.01	0.86–4.68	0.11	---	---	---	1.07	0.45–2.51	0.88	---	---	---	1.25	0.37–4.21	0.72	---	---	---
**Housing Status**																								
Stable	1.00	---	---	---	---	---	1.00	---	---	---	---	---	1.00	---	---	---	---	---	1.00	---	---	---	---	---
Unstable	0.52	0.21–1.27	0.15	---	---	---	1.45	0.67–3.11	0.35	---	---	---	2.23	1.01–4.91	**0.0047**	2.32	0.97–5.55	0.06	0.96	0.33–2.77	0.94	---	---	---
**History of Sex Work**																								
No	1.00	---	---	---	---	---	1.00	---	---	1.00	---	---	1.00	---	---	---	---	---	1.00	---	---	---	---	---
Yes	0.99	0.97–1.02	0.61	---	---	---	1.02	1.00-1.04	**0.04**	1.03	1.00-1.05	**0.03**	1.00	0.98–1.02	0.68	---	---	---	1.01	0.98–1.04	0.59	---	---	---
**Has a Regular Healthcare Provider**																								
No	1.00	---	---	---	---	---	1.00	---	---	---	---	---	1.00	---	---	---	---	---	1.00	---	---	---	---	---
Yes	0.43	0.12–1.58	0.20	---	---	---	1.70	0.65–4.42	0.28	---	---	---	0.5	0.20–1.33	0.16	---	---	---	1.03	0.26–3.98	0.97	---	---	---
**DRUG USE**																								
**Number of Times Overdosed**																								
Continuous	1.10	0.95–1.26	0.21	---	---	---	1.13	0.99–1.29	*0.07*	1.07	0.97–1.19	0.18	1.02	0.98–1.06	0.27	---	---	---	0.88	0.71–1.09	0.25	---	---	---
**Overdosed in Past Year**																								
No	1.00	---	---	---	---	---	1.00	---	---	---	---	---	1.00	---	---	---	---	---	1.00	---	---	---	---	---
Yes	1.15	0.47–2.81	0.76	---	---	---	1.78	0.80–3.95	0.16	---	---	---	1.78	0.81–3.91	0.15	---	---	---	0.70	0.23–2.18	0.54	---	---	---
**Number of Times Witnessed an Overdose**																								
Continuous	1.03	0.99–1.08	0.12	---	---	---	1.01	0.99–1.03	0.58	---	---	---	0.99	0.97–1.01	0.49	---	---	---	0.88	0.79–0.996	**0.04**	0.89	0.79–0.997	**0.04**
**Crack Cocaine Use - Past 30 Days**																								
No	1.00	---	---	---	---	---	1.00	---	---	---	---	---	1.00	---	---	---	---	---	1.00	---	---	---	---	---
Yes	0.90	0.37–2.19	0.82	---	---	---	---	---	---	---	---	---	0.77	0.35–1.67	0.50	---	---	---	0.95	0.32–2.76	0.92	---	---	---
**Benzodiazepine Use - Past 30 Days**																								
No	1.00	---	---	1.00	---	---	1.00	---	---	---	---	---	---	---	---	---	---	---	1.00	---	---	---	---	---
Yes	3.02	1.11–8.21	**0.03**	3.27	1.11–9.65	**0.03**	0.77	0.35–1.67	0.50	---	---	---	---	---	---				2.27	0.78–6.64	0.13	---	---	---
**Pain Medication Use - Past 30 Days**																								
No	1.00	---	---	---	---	---	1.00	---	---	---	---	---	1.00	---	---	---	---	---	---	---	---	---	---	---
Yes	0.49	0.16–1.52	0.22	---	---	---	0.91	0.31–2.66	0.87	---	---	---	2.27	0.78–6.64	0.13	---	---	---	---	---	---	---	---	---
**Heroin or Fentanyl Use - Past 30 Days**																								
No	---	---	---	---	---	---	1.00	---	---	---	---	---	1.00	---	---	1.00	---	---	1.00	---	---	---	---	---
Yes	---	---	---	---	---	---	0.90	0.37–2.19	0.82	---	---	---	2.94	1.08–8.06	**0.04**	3.77	1.21–11.72	**0.02**	0.49	0.16–1.52	0.22	---	---	---
**MOUD USE**																								
**Number of Times on MOUD**																								
Continuous	0.98	0.93–1.04	0.51	---	---	---	1.16	0.99–1.36	*0.07*	1.13	0.96–1.32	0.14	0.96	0.88–1.04	0.33	---	---	---	0.81	0.61–1.07	0.13	---	---	---
**MOUD Type**																								
Buprenorphine	1.00	---	---	1.00	---	---	1.00	---	---	1.00	---	---	1.00	---	---	1.00	---	---	1.00	---	---	1.00	---	---
Methadone	6.19	1.97–19.51	**0.002**	6.61	2.03–21.56	**0.002**	0.94	0.43–2.03	0.87	0.82	0.32–2.14	0.69	1.30	0.60–2.84	0.50	1.10	0.43–2.86	0.84	0.78	0.26–2.33	0.66	0.69	0.22–2.19	0.53

aOR = Adjusted Odds Ratio; CI = Confidence Intervals; P = P-Value; Bolded values = significant at p < 0.05; Italicized P-Values = p < 0.10.

***Note.*** Naltrexone was excluded from the models as the small cell sizes precluded multivariable analyses. Variables with a p-value of < 0.10 in bivariate analyses were included in the multivariable model. Since older individuals have more years to use drugs and MOUD than younger individuals, all multivariable models adjusted for age.

In the qualitative interviews, participants on MOUD contextualized the quantitative findings by providing insights into their co-use of heroin/fentanyl and MOUD. Several participants, including the following female participant, reported that their dose of methadone was too low to satisfy their physiological cravings for heroin/fentanyl, which contributed to their ongoing concomitant use.

*I was on [methadone] as low as 30 and sustained on a 30 mg dose, but I would suffer through that and just tell myself, “It’s all in your head. You’re not sick. You’re not sick.” [Increasing my dose,] I feel like it would help me more with the cravings.****–*Currently Prescribed Methadone, Medium City**

Other participants on either methadone or buprenorphine reported that while MOUD helped manage their physical craving for heroin/fentanyl, psychological cravings and environmental triggers persisted. Some participants indicated that these triggers contributed to their ongoing use of heroin/fentanyl. For example, one male participant noted:

*[Methadone], it’s definitely changed a lot of things. I don’t wake up sick anymore. I can go and get my dose in the morning and I don’t have to get high. I don’t need to do heroin or fentanyl. I don’t need to, but there’s something in my mind that still tells me I want to do it. And then when I see people do it, it makes me in my mind want to do it even more. ***– Currently Prescribed Methadone, Medium City**

#### Drug use & MOUD history: crack cocaine use

As shown in Table 1, more than half the sample of people who use drugs used crack cocaine in the past 30 days (52.6%). In examining the association between crack use and MOUD history among the full sample (N = 284), the age-adjusted, multinomial logistic regression model (Table 2) showed that past 30-day crack cocaine use was significantly associated with the higher odds of being prescribed MOUD in the past (aOR = 2.73; 95% CI = 1.36–5.46; *p* = 0.005) and currently (aOR = 3.05; 95% CI = 1.49–6.16; *p* = 0.002). When restricting the sample to only those on methadone or buprenorphine (N = 108), multivariable logistic regression analyses (Table 3) showed that living in a medium city (ref: small city or town; aOR = 2.89; 95% CI = 1.04–8.03; *p* = 0.04) and having a history of sex work (aOR = 1.03; 95% CI = 1.00-1.05; *p* = 0.03) were each associated with the increased odds of past 30-day crack cocaine use.

Qualitative findings helped to explain why crack use was more prevalent among those with a past or current history of MOUD use as opposed to those who had never taken MOUD. Specifically, many participants discussed a change in their drug of choice after initiating MOUD. Indeed, numerous participants indicated that MOUD blocked their cravings for opioids yet made them feel tired at times. As a result, many participants started using or increased their use of powdered or crack cocaine after initiating MOUD to provide them with energy to get through their day. For example, one female participant from a medium city reported, “*The methadone, it like makes you crave cocaine, you know what I mean, because it brings you down, so now you want that up, so then you tend to do more coke.”* Another female participant who was previously dependent on prescription opioids, reported using powdered and crack cocaine for energy while taking buprenorphine. She said:

*When I first started getting the Suboxone I used to get the same kind of like energy that I got when I was taking the pills. You do not get a high or like a body high where you feel good all over. I just got a little like coffee, like I had a good coffee and I got some energy. But I don’t really get that anymore. I’ve been on them so long… But it’s a hell of a lot better than having to wake up and get high [on opioids] and do what you got to do to get money, steal, sex, whatever, to get high.’ I’d rather be on a clinic and do a urine. And I’m honest. [I tell the clinic staff], ‘Hey, I did some coke. I’m going to be dirty for coke.’ –***Currently Prescribed Buprenorphine, Small City or Town**

Although many participants reported that MOUD was effective in managing their cravings, some participants still wanted to get high, and so they increased or, in some cases, replaced their use of heroin/fentanyl with other drugs such as powdered and crack cocaine. For example, one male participant who was formerly prescribed methadone and one female participant who is currently prescribed methadone reported:

*I kind of used [methadone] for a little while then like…that’s actually when I really started using cocaine because before that, it was like a once in a while type of thing. Like if it was around, I would do it. Like I never searched it, sought it out. But yeah, once I got on the methadone, and like I realize that I wasn’t gonna get sick and I didn’t have to spend all my money on opiates then like I kind of started experimenting with other things. –* **Formerly Prescribed Methadone, Medium City**

*I got on the Vivitrol shot. It was actually good but for some freaking reason, my dumbass decided to start smoking crack and then I turned. I feel like I literally found her substitute. Like crack was my life. I was sectioned, I came out, I smoked crack, I slept with it, I would wake up before I even peed, I smoked it. Yeah, I don’t know why, but I wanted to have it. It was great. –* **Currently Prescribed Methadone, Formerly Prescribed Naltrexone, Medium City**

Qualitative interviews also supported the findings linking crack to sex work among people on MOUD. In the context of the energy-dampening effects of methadone, several participants talked about using crack to be able to function and work. Additionally, several participants reported engaging in sex to “feed” their crack addiction, with many having been paid for sex with crack. For example, one female participant who currently uses methadone and is from a medium city noted, “*I’ve done sex for crack many times. I did sex for crack yesterday. You know, I had him feeding me crack all night*.”

#### Drug use & MOUD history: benzodiazepine use

Benzodiazepines were used by about a third of the sample in the past 30 days (30.3%; Table 1). In the age-adjusted multinomial logistic regression model examining the association between current benzodiazepine use and current and past history of MOUD use among the full sample (N = 284), past 30-day benzodiazepine use was associated with the increased odds of being currently prescribed MOUD (aOR = 2.71; 95% CI = 1.22–6.01; *p* = 0.01). Further, in the multivariable logistic regression model (Table 3) examining factors associated with benzodiazepine use among those on MOUD (N = 108), past 30-day heroin/fentanyl use was significantly associated with the increased odds of past 30-day benzodiazepine use (aOR = 3.77; 95% CI = 1.21–11.72; *p* = 0.02).

In qualitative interviews, many participants reported using both prescribed and street-acquired benzodiazepines to manage life stressors as well as treat their mental health symptoms. One male participant on methadone from a large city reported being prescribed benzodiazepines for clinically-diagnosed mental health conditions and sought to correct the misconception that people who take benzodiazepines are “addicts.” He noted, “*I go to get my doses—I’m prescribed benzos. That doesn’t mean I’m a drug addict because I’m prescribed Klonopin because I have panic attacks*.” Not all participants, however, reported being able to access benzodiazepines to treat their anxiety, leading some to take street-acquired benzodiazepines, sometimes with adverse consequences. For example, one woman from a medium city reported that the use of street-acquired benzodiazepines contributed to her discontinuation of methadone: “*I was on the methadone clinic and then I got kicked off. Because of my anxiety, I was with my ex and he gave me Xanax, so I got kicked off the clinic because of that.”*

Not all participants, however, reported using benzodiazepines to manage their mental health symptoms. For example, one male participant from a small city or town reported using benzodiazepines to enhance the effects of methadone (i.e., “to get high”): “[*While on methadone, I used] benzos…cause they mix well with the methadone. They intensify everything*.”

#### Drug use & MOUD history: pain medication use

Less than a fifth of the sample (18.1%) reported using prescribed or street-acquired pain medication in the past 30 days (Table 1). Nearly half of those with a history of prescription pain medication use (47.8%) reported having received a fake pain pill on the street, and 46.1% reported that it was hard to find a genuine pill on the street compared to the previous year. Additionally, 84.4% reported that it was hard or extremely hard to obtain prescription pain medication from a doctor.

In age-adjusted multinomial regression analyses (Table 2) with the full sample (N = 284), past 30-day pain medication use was associated with the decreased odds of being currently prescribed MOUD (aOR = 0.31; 95% CI = 0.13–0.73; *p* = 0.008). When restricting the sample to only those on MOUD (N = 108; Table 3), multivariable logistic regression analyses found that as the number of witnessed overdoses increased, the odds of pain medication use decreased (aOR = 0.89; 95% CI = 0.78–0.99; *p* = 0.04).

In qualitative interviews, many participants attributed the low use of prescribed or street-acquired pain medication to the fact that fewer physicians are prescribing pain medication to people these days, particularly individuals with a substance use disorder. Although some participants reported acquiring pain medication from the street, many people recognized that street-acquired pills were often fake and contained fentanyl. Further, several participants, particularly those who had witnessed a lot of fatal overdoses, reported avoiding the use of street-acquired pain medication to reduce their risk of overdose. For example, one female participant from a medium city noted, “*You can tell Perc-30s if they’re real. You put ‘em on tin foil and you light underneath it. If they slide, that means they’re real. [Otherwise,] you don’t go to them no more, you know? But there are a lot of pills [they’re] making Fentanyl with….[so] that’s a wrap [for me]*!”

Additionally, in interviews, several participants in the sample reported being prescribed buprenorphine for pain. For example, one female participant from a medium city noted, *“Some doctors give [buprenorphine] to you for pain. I know a couple people who have it for pain.”* Similarly, one male participant from a small town noted, *“I’ve been taking subs for my pain, the pain center put me on ‘em… I’ve had 18 surgeries.”* Several participants explicitly linked their use of MOUD to a reduction in prescribed and street-acquired prescription pain medication use as these medications, particularly buprenorphine, not only helped them to reduce their use of illegal opioids but, in some cases, also helped them to manage their physical as well as emotional pain. For example, one female participant from a small city initially became dependent on prescription opioids and later switched to heroin. She noted self-medicating with prescription opioids and heroin but reducing her use when she was prescribed buprenorphine. She noted, “*So, my mom had died the year before and I was just depressed, and that’s why I [took pain pills and later heroin], you know? That’s why…It killed the pain, but Subs do work. They keep you off of it, you know*?”

## Discussion

In this Massachusetts-based, mixed-methods study of people with past 30-day illegal drug use, MOUD use was high, with 79.9% reporting a history of MOUD use in their lifetime and 28.7% who were currently on MOUD (56.4% buprenorphine; 41.8% methadone; 1.8% naltrexone). Past 30-day drug use was also high among the full sample, with 74.4% reporting the use of heroin/fentanyl in the past 30 days, 51.4% crack cocaine, 31.3% benzodiazepines, and 18% pain medications. Although many participants in qualitative interviews reported reducing and even ceasing their use of illegal opioids while on MOUD, inadequate dosage, untreated trauma, psychological cravings, and economic and environmental factors served as triggers for the continued use of heroin/fentanyl and other drugs. Findings from this study extend prior quantitative and qualitative research with people who use drugs in North America and have implications for the effective treatment of OUD in clinical settings [14–17, 19–22, 25, 26, 29, 30, 34].

Notably, heroin/fentanyl use was high among this sample of people who use drugs, with multivariable models showing that participants on methadone had almost five times increased odds of concurrently using heroin/fentanyl than those on buprenorphine. These findings are not surprising given that buprenorphine-naloxone and naltrexone are partial and full opioid antagonists, respectively, and can trigger withdrawal symptoms in people who use other opioids [10]. In qualitative interviews, participants often reported intentionally choosing methadone over naltrexone as it allowed them to continue to use illegal opioids. For some individuals, their current MOUD dosage was too low to help them manage their physiological cravings – a finding that aligns with clinical evidence on the need for higher MOUD doses to manage withdrawal symptoms among people who use fentanyl [43]. Even when MOUD doses were sufficient enough to block the physiological cravings for opioids, environmental triggers, trauma, and untreated mental health issues drove people to self-medicate with illegal substances, including opioids and other drugs. As described in the literature, pre-existing mental health symptoms and trauma may drive the use of illegal drugs as a means of coping; however, in other cases, trauma and poor mental health may emerge as a result of chronic OUD and its social sequelae [44–46]. For example, many people who use drugs, including participants in the present study, reported witnessing the overdose deaths of friends or loved ones as well as encountering violence in their everyday lives [47, 48]. Thus, for many people who use drugs, if they did not have a history of trauma before using drugs, the common and frequent exposure to traumatic events can induce adverse mental health issues that can complicate recovery efforts and lead to relapse [47].

The use of past 30-day prescribed- and street-acquired benzodiazepines was elevated among the full sample, with the multinomial logistic regression model showing that those who had recently used benzodiazepines had almost three times increased odds of currently using MOUD as opposed to never using MOUD. Further, logistic regression analyses showed that among people on buprenorphine or methadone, benzodiazepine use was positively associated with recent heroin/fentanyl use. In qualitative interviews, many participants reported taking benzodiazepines with and without a prescription to manage their anxiety as well as to enhance the euphoric effects of MOUD, particularly methadone. Research shows that people with OUD often have comorbid mental health symptoms [49, 50], and benzodiazepine use is commonly prescribed to treat anxiety, panic disorders, and even sleep disturbances among people with OUD [32, 51]. Further, in prior qualitative research with people with a lifetime history of non-medical opioid use [33], participants reported using benzodiazepines with and without a prescription to manage their untreated mental health symptoms [32, 33] and enhance the effects of opioids and other drugs [33]. Although the use of benzodiazepines together with illegal opioids and/or methadone or buprenorphine is associated with elevated risk for overdose [18, 26–28, 52], and people on MOUD are often aware of these risks [32], national OUD treatment guidelines acknowledge that untreated OUD carries a greater risk of morbidity and mortality than does the co-use of benzodiazepines and MOUD and calls for clinicians to carefully manage these prescriptions [52–54]. Given the overall benefit of MOUD [10, 52], findings from this study, together with prior research, underscore the importance of providers being aware of the drivers of concurrent benzodiazepine use among MOUD patients and the need for trauma-informed MOUD care and access to mental health services for people with OUD.

Although heroin/fentanyl was the most commonly used drug among the full sample and those on MOUD, past 30-day crack cocaine use was also endorsed by more than half the sample, and mixed-methods findings revealed interesting shifts in the use of powdered and crack cocaine following MOUD uptake. Specifically, in the multinomial logistic regression model, past 30-day crack cocaine use was associated with the increased odds of using MOUD currently and in the past as opposed to never. Consistent with prior qualitative research with people on methadone in Vancouver, Canada [34], our quantitative findings were contextualized in qualitative interviews in which participants described increasing their use of powdered and crack cocaine while on MOUD to counteract the energy-dampening effects of MOUD, particularly methadone. Additionally, quantitative findings demonstrated an association between sex work and crack use, and qualitative findings showed that many participants who engaged in sex work were often paid with crack. Moreover, several participants reported using crack to give them the energy to work – a finding that aligns with a 2013 qualitative study conducted with street-based female sex workers who use drugs in Vancouver, Canada, prior to the widespread availability of fentanyl in the drug supply [55]. Additionally, in qualitative interviews, participants acknowledged that MOUD was largely effective in managing their opioid cravings and withdrawal symptoms, yet, extending prior research with people on methadone from Vancouver, Canada [34], some participants reported turning to powdered and crack cocaine to fulfill their desire to change their consciousness and experience a euphoric high. These findings highlight the psychological, physiological, and economic drivers of concurrent crack and MOUD use among people who use drugs.

Notably, past 30-day pain medication use was endorsed by less than a fifth of the sample, and multinomial logistic regression analyses showed that those who had recently taken prescribed or street-acquired pain medication had reduced odds of currently using MOUD as opposed to never having used MOUD. Although there was no significant difference in the odds of recent pain medication use among those on methadone vs. buprenorphine, as the number of overdoses that participants witnessed increased, the odds of using prescribed or illegal pain medication in the past 30 days decreased. Further, in qualitative interviews, participants reported that genuine prescription pain medication was harder to come by on the street and through a doctor, with nearly half of those with a history of pain medication use reporting having received a fake pill on the street. This finding aligns with data showing a decrease in opioid pain medication prescribing [56] due in part to stricter prescribing guidelines in the wake of the prescription opioid epidemic [57, 58]. Further, in qualitative interviews, participants reported that many prescription pain pills on the street contain fentanyl – a finding that aligns with DEA drug seizure data [59]. It is possible that those who had witnessed more overdoses were less likely to have recently used pain medication out of concern for the high risk of overdose when using street-acquired pain medications that contain fentanyl [60, 61] as well as an inability to access genuine opioid pain medication via a doctor or on the street [56, 62]. Notably, in alignment with the literature [63–65], several participants reported that buprenorphine was effective in helping participants to manage opioid cravings as well as pain, which may also explain the lower prevalence of pain medication use as opposed to the use of other drugs among the people who use drugs who were sampled.

Despite the fact that the heroin supply has been contaminated with fentanyl for many years [48, 66, 67], recent data show that the stimulant and counterfeit pill supplies are now also being contaminated with fentanyl [36, 59, 68–70], The rise in the fentanyl-contaminated stimulant and counterfeit pill supply has led to the unintended consumption of fentanyl and an increase in opioid overdoses among people who use stimulants and pressed pills [36, 59, 68, 69, 71]. Research conducted in Canada has shown that providing access to supervised consumption sites and a safe drug supply, including heroin-assisted treatment and injectable or tablet formulations of opioid agonist treatments, has been linked to reductions in overdose deaths [72–74] and improved treatment outcomes [75], health, quality of life, and social functioning [76]. However, as of 2022, safe consumption sites are illegal in all but one US state (i.e., Rhode Island) [77] and efforts to increase access to a safe drug supply continue to be thwarted across the US. As such, people with OUD in the US. have primarily relied on FDA-approved forms of MOUD, such as liquid opioid agonists (i.e., methadone) and partial and full opioid antagonists such as oral and injectable buprenorphine-naltrexone and injectable naltrexone, respectively [10]. Notably, in the US all of these medications are subject to restrictive dispensing policies, particularly methadone, which typically requires witnessed ingestion, supervised urine toxicology screening, and zero-tolerance policies that, despite national guidelines, can lead to administrative discharge for individuals found to be using illicit substances while receiving methadone [10, 29].

In addition to being a justification for administrative discharge, the concurrent use of heroin/fentanyl and other drugs among those on MOUD has historically been considered a treatment failure by many clinicians [19, 54]. Yet, research shows that individuals who use MOUD are at decreased risk of experiencing a fatal overdose when intentionally or unintentionally exposed to fentanyl in the drug supply [14, 15, 17]. Additionally, current national OUD practice guidelines state that co-occurring drug use should not be used as a reason to withhold or terminate MOUD treatment [52–54]. Instead, OUD treatment guidelines call for a more intensive treatment approach for people who concurrently use MOUD and one or more illegal drugs [52–54]. Further, although as of June 2021 Massachusetts is one of only two states in the nation to prohibit administrative discharge for not being abstinent from opioids [78], similar to MOUD patients in prior national qualitative research [30], participants in this study reported that the concurrent use of any illegal drug and MOUD was often the reason for MOUD administrative discharge in outpatient treatment programs. Although it is possible that participants were administratively discharged before the passage of the Massachusetts policy, it is also possible that clinics and individual providers in Massachusetts do not adhere to administrative discharge prohibition policies for patients engaging in ongoing illegal drug use [29, 30, 79]. Regardless, the forced discontinuation of MOUD by treatment providers can lead to deadly consequences as individuals with a reduced tolerance for fentanyl and who are no longer on MOUD are at excess risk for fatal overdose [14, 17, 31].

The prevalent concurrent use of illegal drugs and MOUD among this sample, the potential for administrative discharge from outpatient programs for people concurrently using illegal drugs and MOUD, and research linking MOUD use to reduced opioid fatality risk [14, 15, 17] underscore the need for a paradigm shift from the rigid “no tolerance” MOUD prescribing practices of the past to a more harm reduction-focused model that recognizes that recovery is a continuum. To that end, research in Canada and the US finds that flexible MOUD treatment models that allow for take-home dosing of methadone and buprenorphine-naltrexone are feasible, acceptable, safe, effective, and, in some cases, associated with better treatment outcomes than standard models of care [63, 80–82]. In addition to providing more flexibility, expanded treatment models should also account for the impact of trauma on drug use behaviors and use a trauma-informed approach to treatment [44] that leverages and seeks to build internal and external patient resources [83] such as distress tolerance [84, 85] and social support [86] to reduce ongoing drug use and help patients achieve their recovery goals. Flexible treatment models and clinical practices that account for the whole person are urgently needed to prevent overdoses and save the lives of people with OUD in the age of fentanyl.

### Limitations

Several methodological limitations should be considered in light of our findings. Given that we conducted a cross-sectional study, causality cannot be inferred. We did not quantitatively assess prescribed vs. non-prescribed benzodiazepine or pain medication use, though we were able to explore this qualitatively. Additionally, we explored MOUD and drug use among people who used illegal drugs in the past 30 days in Massachusetts. Thus, our findings may not be generalizable to individuals living in other regions of the US and are not representative of all people on MOUD. Finally, we tended to recruit individuals living with more advanced substance use disorders as well as low-income, marginally-housed people. Future research should aim to recruit a diverse sample of people on MOUD, including those with no recent drug use, those who are stably housed, and those with higher incomes, to fully characterize the prevalence of concurrent drug use and barriers and facilitators to achieving long-term recovery among the entire cross-section of people on MOUD.

## Conclusion

Through our novel mixed-methods study, we documented the persistence of drug use and prescribed medication therapy as well as potential reasons for concurrent drug and MOUD use among people who use drugs with and without a history of MOUD use. Findings underscore the need for MOUD providers to understand the physiological, psychological, economic, and environmental drivers of concurrent drug use among people on MOUD and ensure proper dosing and access to trauma-informed MOUD care and mental health services for this population.

## Data Availability

The datasets used and/or analyzed during the current study are available from the corresponding author on reasonable request.

## List of Abbreviations

OUD: Opioid Use Disorder
MOUD: Medications for Opioid Use Disorder
